# The Association between COVID-19 Related Anxiety, Stress, Depression, Temporomandibular Disorders, and Headaches from Childhood to Adulthood: A Systematic Review

**DOI:** 10.3390/brainsci13030481

**Published:** 2023-03-12

**Authors:** Giuseppe Minervini, Rocco Franco, Maria Maddalena Marrapodi, Vini Mehta, Luca Fiorillo, Almir Badnjević, Gabriele Cervino, Marco Cicciù

**Affiliations:** 1 Multidisciplinary Department of Medical-Surgical and Odontostomatological Specialties, University of Campania “Luigi Vanvitelli”, 80121 Naples, Italy; 2 Department of Biomedicine and Prevention, University of University of Rome “Tor Vergata”, 00100 Rome, Italy; 3 Department of Woman, Child and General and Specialist Surgery, University of Campania “Luigi Vanvitelli”, 80130 Naples, Italy; 4 Associate Professor, Department of Public Health Dentistry, Dr. D. Y. Patil Dental College and Hospital, Dr. D. Y. Patil Vidhyapeeth, Pimpri, Pune 411045, India; 5 Department of Biomedical and Dental Sciences and Morphofunctional Imaging, School of Dentistry, University of Messina, Via Consolare Valeria, 1, 98125 Messina, Italy; 6 Verlab Research Institute for Biomedical Engineering, Medical Devices and Artificial Intelligence, 71000 Sarajevo, Bosnia Erzegovina; 7 Department of General Surgery and Medical-Surgical Specialties, School of Dentistry, University of Catania, 95124 Catania, Italy

**Keywords:** COVID-19, temporomandibular disorders, TMJ, TMD, temporomandibular joint, bruxism

## Abstract

**Objective:** The coronavirus belongs to the family of Coronaviridae, which are not branched single-stranded RNA viruses. COVID-19 creates respiratory problems and infections ranging from mild to severe. The virus features mechanisms that serve to delay the cellular immune response. The host’s response is responsible for the pathological process that leads to tissue destruction. Temporomandibular disorders are manifested by painful jaw musculature and jaw joint areas, clicks, or creaks when opening or closing the mouth. All these symptoms can be disabling and occur during chewing and when the patient yawns or even speaks. The pandemic situation has exacerbated anxieties and amplified the vulnerability of individuals. Therefore, from this mechanism, how the COVID-19 pandemic may have increased the incidence of temporomandibular disorders is perceived. The purpose of this review is to evaluate whether COVID-19-related anxiety has caused an increase in temporomandibular dysfunction symptoms in adults to children. **Methods:** PubMed, Web of Science, Lilacs, and Scopus were systematically searched, until 30 July 2022, to identify studies presenting: the connection between COVID-19 with temporomandibular disorders. **Results:** From 198 papers, 4 studies were included. Literature studies have shown that the state of uncertainty and anxiety has led to an increase in the incidence of this type of disorder, although not all studies agree. Seventy-three studies were identified after viewing all four search engines; at the end of the screening phase, only four were considered that met the PECO, the planned inclusion, and the exclusion criteria. All studies showed a statistically significant correlation between temporomandibular disorders and COVID-19 with a *p* < 0.05. **Conclusions:** All studies agreed that there is an association between COVID-19 and increased incidence of temporomandibular disorders.

## 1. Introduction

The coronavirus belongs to the family of Coronaviridae, which are not branched single-stranded RNA viruses. COVID-19 creates respiratory problems and infections that range from mild to severe [1,2,3]. During the 2000s, two viruses of animal origin were caused by species shifting, the severe acute respiratory coronavirus syndrome (SARS-CoV-1) and the Middle East respiratory coronavirus disease (MERS-CoV) [4,5,6,7,8,9]. Coronaviridae belongs to the Betacoronavirus genus, group 2, which causes serious diseases. After several years the virus mutated, reported for the first time in humans in 2019, in the city of Wuhan. The virus consists of 16 proteins, all of which are used for replication. The virus has become so widespread that on 11 March, the World Health Organization declared a pandemic. SARS-CoV-2 has a genomic sequence that shares 80% similarity to SARS-CoV-1 and 50% to MERS-CoV [10,11,12,13,14]. The spike protein is an important structural protein that allows the virus to enter the host’s cells and is also highly variable. The virus binds to the angiotensin-converting enzyme 2 (ACE2). Therefore, all cells expressing this receptor are potentially susceptible to this infection. The study by Zo et al. evaluated the expression of the ACE 2 receptor and, thus, created a risk map of different human tissues. All the following tissues are at high risk: lung, heart, esophagus, kidney, bladder, and ileum. Furthermore, in the oral cavity, especially in the epithelial cells of the oral mucosa and tongue, an elevated expression of ACE2 was observed [15,16,17,18,19,20,21,22,23]. The virus features mechanisms that serve to delay the cellular immune response. The host response is responsible for the pathological process that leads to tissue destruction. In some cases, the immune response leads to the resolution of the infection. In other cases, the secondary immune response leads to the destruction of the individual’s tissues and causes lung damage, acute respiratory distress syndrome, respiratory failure, shock, organ failure, and possible death. COVID-19 triggers hypercoagulation and causes neurological damage, and, in severe cases, leads to death. Fever, cough, runny nose, nasal congestion, anosmia, dysgeusia/hypogeusia, diarrhea, and nausea/vomiting are the most typical symptoms [24,25]. Fatigue, eye discomfort (conjunctival discharge), and arrhythmias are other clinical signs. Less common symptoms are gastrointestinal problems. Acute cholestasis and pancreatitis occur more frequently in adolescent patients.

The severity of the symptoms depends on the state of the infection [26]. In the case of preclinical symptoms, a diagnosis can be made through thoracic CT from which subclinical lung lesions are shown. In severe situations, oxygen saturation may drop below 92%. Respiratory failure, shock, encephalopathy, myocardial damage or heart failure, coagulation malfunction, acute renal injury, and multiorgan dysfunction occur, as the infection and damage worsen [27].

Temporomandibular disorders are characterized by jaw joint and jaw muscle soreness, clicks, or creaks when opening or closing the mouth [4,5,28,29,30,31]. All these symptoms can be disabling and occur during chewing and when the patient yawns or even speaks [32,33,34,35]. Temporomandibular disorders are also manifested by pain in the cervical area, frequent headaches, and pain in the dental arches, and are caused and worsened by stress and psychosocial problems [36]. The pandemic has led to a deterioration in social and living conditions and has become one of the main public health emergencies that exacerbated anxieties and amplified the vulnerability of individuals [37,38,39,40,41]. Therefore, from this mechanism, how the COVID-19 pandemic may have increased the incidence of temporomandibular disorders is perceived. Indeed, this sense of uncertainty and a lack of confidence in medical therapy and in the various governments to stem the pandemic situation has exacerbated the incidence of COVID-19 [42,43]. Psychosocial and uncertainty factors can lead to additional adrenocortical steroid release and lead to peripheral vasoconstriction, which causes sleep sensations such as hot and cold sweats, palpitations, rapid heartbeat, nausea, abdominal pain, diarrhea, and constipation. Incidence studies have reported an increase in bruxism and joint pain, all due to increased uncertainty, and stress caused by job loss or separation from family members [44,45,46]. In fact, the increased stress, anxiety, and depression induced by the pandemic situation have increased the incidence of symptoms of joint disorders [46]. On the other hand, people with temporomandibular disorders are more susceptible to anxiety due to COVID-19 and increased joint pain [47]. Several clinical studies have evaluated how the lockdown situation has amplified the symptoms of temporomandibular disorders. Therefore, the importance of this review, in addition to confirming that there is a correlation between anxiety and COVID-19 stress, has an important clinical implication in that the treatment of these patients requires a multidisciplinary approach (dentist, psychologist, and physician) in order to properly treat this condition. The primary purpose of this systematic review was to analyze the major literature studies and see how anxiety and stress, caused and increased by COVID-19, cause an increase in temporomandibular dysfunction symptoms, such as headaches. Moreover, as a secondary outcome, we wanted to evaluate how patients already suffering from TMD experienced worsening symptoms. In addition, we wanted to evaluate how clinicians and dentists should deal with this problem during their work [48].

## 2. Materials and Methods

### 2.1. Eligibility Criteria

The following criteria were used to determine the admissibility of each document: Population (including animal species), exposure, comparator, and outcomes (PECO) [49].

(P) Participants consisted of patients.
(E) The exposure consisted of patients with a diagnosis of TMD and evaluated with DC-TMD at the time of COVID
(C) The comparison was patients in pre-COVID times with TMD.
(O) The outcome consisted of assessing the prevalence of temporomandibular disorders in patients at the time of COVID. The secondary outcome consisted of assessing the correlation between TMD prevalence and severity during the time of COVID, compared with the time before COVID. Although the correlations between TMD, stress, and preoccupation due to the lifestyle changes from COVID were evaluated. Therefore, the secondary purpose was also to evaluate the correlation between COVID-19-related anxiety, stress, and increased TMD symptoms.

Exclusion criteria were: (1) People with a history of TMJ trauma; (2) people with any inflammatory problems or rheumatic diseases (such as, psoriatic arthritis or rheumatoid arthritis); (3) people with fibromyalgia; (4) people with congenital abnormalities or neoplastic conditions in the TMJ region; (5) studies that were written in a language other than English; (6) an absence of the full text (posters and conference abstracts); (7) animal studies; (8) case reports; (9) review articles.

### 2.2. Search Strategy

**Figure 1:**
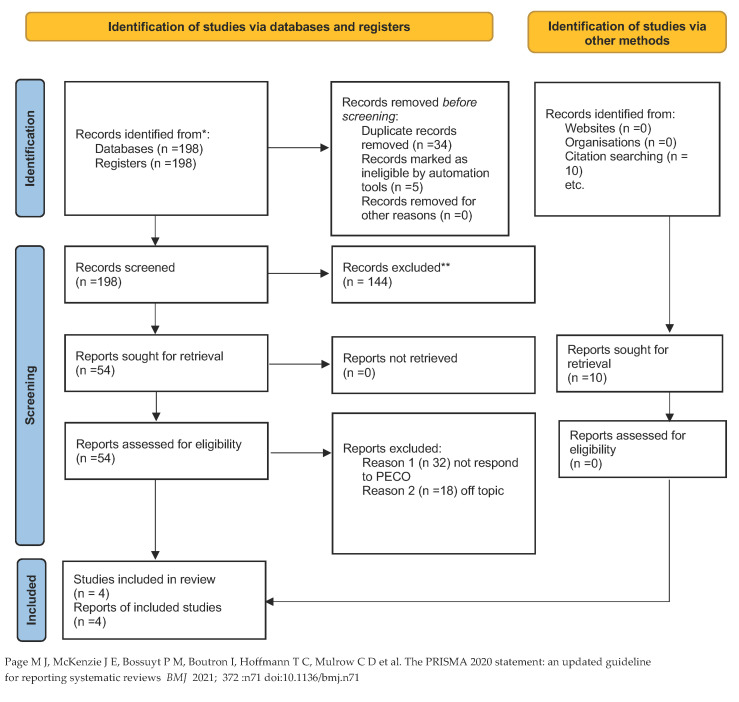
PRISMA flowchart [50]. * excluded because they are review.

Authors systematically explored the databases of PubMed, Web of Science, Lilacs, and Scopus for published papers from 2019 until 30 September 2022, according to each specific thesaurus and following the strategy. The term “COVID-19” has been combined with “Temporomandibular disorders”. During the literature search using the Boolean NOT operator, case reports were excluded from the beginning. The web search was assisted using MeSH (Medical Subject Headings) (Table 1). The criteria for this review are described in the PRISMA and by the following flowchart (Figure 1). Additionally, a manual search of earlier systematic reviews on the same subject was performed in the sources. The Cochrane Handbook for Systematic Reviews of Interventions and Preferred Reporting Items for Systematic Reviews (PRISMA) criteria were followed in conducting this systematic review. The International Prospective Register of Systematic Reviews (PROSPERO) has recorded the systematic review protocol under the accession number CRD42022327431.

### 2.3. Data Extraction

Two reviewers (G.M. and R.F.) independently extracted data from the included studies using a customized data extraction on a Microsoft Excel sheet. In case of disagreement, a consensus was reached through a third reviewer (M.C.).

The following data were extracted: (1) first author; (2) year of publication; (3) nationality; (4) type of study; (5) classification of TMD; (6) sample size; (7) age of study participants; (8) main findings; (9) differences in symptom severity before and after the Covid era.

### 2.4. Quality Assessment

The risk of bias in the papers was assessed by two reviewers using Version 2 of the Cochrane risk-of-bias tool for randomized trials (RoB 2). Any disagreement was discussed until a consensus was reached with a third reviewer.

## 3. Results

### 3.1. Study Characteristics

At the end of the research, 198 studies were identified from the search conducted on the 4 engines. During the initial phase, 34 items were excluded because they were duplicates, and 5 because they were not in English. During the initial screening phase, 144 articles were excluded from the search engines because they did not meet the inclusion criteria or were reviews. During the final screening phase, the abstracts of 54 articles were evaluated, and whether they met the inclusion criteria and the PECO. Only four were chosen to be included in the present systematic study, as illustrated by the PRISMA 2020 flowchart in Figure 1. A total of 50 articles were excluded: 21 were off-topic, and 29 did not respond to the PECO questions. In addition, 10 articles were manually searched for in the bibliographies of the studies; however, they were not excluded as duplicates.

According to the PECO model, the remaining papers were chosen for the title and abstract screening. The residual papers were also selected for title and abstract screening. Finally, four articles were presented in the publication on the search engines used. The studies considered have a timeframe from 2020 to 2022. The studies analyzed were conducted in various parts of the world: Brazil, Italy, Israel, and the USA. A total of 556 subjects were analyzed. Regarding the study design, there were two randomized clinical trials, one retrospective study, and one prospective cohort study. Among these studies, all used the DC/TMD criteria. All studies compared the duration and severity of symptoms before and after COVID through stress scale ratings, all of which differed among the studies analyzed.

#### Main Findings

The study of Arias [51] is a retrospective study. The study included 288 patients who underwent a history and examination of the temporomandibular joint to assess the presence of symptoms, according to TMD diagnostic criteria. The study examined and analyzed two groups of patients: group 1 consisted of 108 patients who were visited and evaluated in the pre-covid era and group 2 consisted of 180 patients who were visited and evaluated in the COVID-19 era. The findings of this investigation revealed a considerable rise in parafunction in men and women with a significance of *p* < 0.001. The presence of diurnal and nocturnal bruxism was more common during COVID-19, exclusively in women AB-*p* < 0.001; SB *p* = 0.014. [51]. The study of Visco [52] evaluated 182 patients who underwent Axis II. The questionnaire assessed the onset of TMD symptoms and perceived stress levels. Axis II of the DC/TMD is a questionnaire that evaluates chronic jaw pain, the impediment on the social life that follows the pain, and the evaluation of psychosocial stresses. Axis II of the DC/TMD combines joint pain and the psychosocial stresses that follow. Analysis of the questionnaire results revealed that 40.7% of the subjects complained of TMD symptoms in the last month. While 60.8% of them reported facial pain in the past 3 months. A total of 51.4% said symptoms worsened in the last month and 51.4% of those subjects reported that their symptoms had worsened due to the lockdown [52]. The study of Mendonca et al. [53] evaluated the intensity and quality of life in a group of women with temporomandibular disorders, assessing their pain and the intensity of the disorders, before and during the pandemic period. In this study, women with TMD were evaluated and their pain levels and oral health-related quality of life (OHRQoL) were compared before (T1) and during (T2) the COVID-19 pandemic. The study examined a group of 41 women with temporomandibular dysfunction symptoms. The subjects were submitted to the questionnaire of the Oral Health Impact Profile-14 (OHIP-14) and tested to evaluate the joint pain scale, in order to assess the quality of life of these patients with temporomandibular dysfunction. The data were collected in two stages. At time T1, before the pandemic, and at time T2, during the pandemic period. Statistical analysis was performed to a 5% significance threshold (Wilcoxon, chi-square or Fisher test, multiple linear regressions). No statistically significant differences were found in pain intensity (*p* = 0.26) and overall OHIP-14 scores (*p* = 0.53) [53]. The study of Asquini et al. [54] evaluated the impact of COVID-19 on the psychology and severity of facial pain in patients with temporomandibular dysfunction. A total of 45 adults were enrolled, including 19 with chronic TMD and 26 with acute/subcutaneous TMD. The examination and symptom assessments were carried out before the pandemic and during the lockdown period, and follow-ups were performed. COVID Stress Scales (CSS) have been created to measure the extent of distress. Scores on the scale were notably greater in patients with chronic TMD (*p* < 0.05). The change in patients with chronic disease was statistically correlated with the change in CSS scores (r = 0.59; *p* = 0.017) [54] (Table 2).

### 3.2. Quality Assessment and Risk of Bias

**Figure 2:**
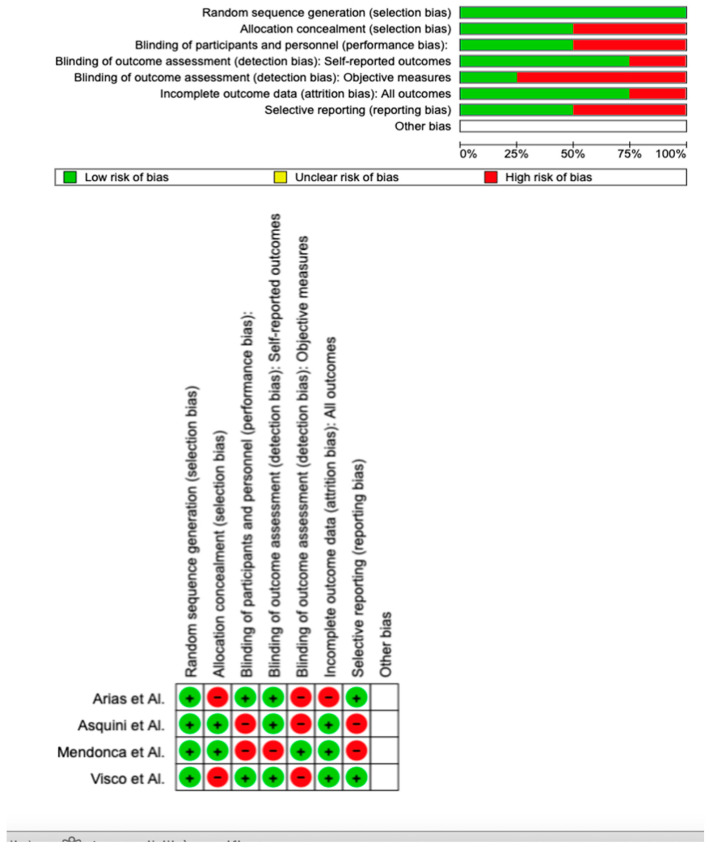
Risk of bias [51,52,53,54]. Green are low risk of bias; red are high risk of bias.

Using RoB 2, the risk of bias among the analyzed studies was estimated and reported in Figure 2. Regarding the randomization process, 100% of the studies ensured a low risk of bias. However, 50% of the studies excluded a performance bias, although 75% reported all outcome data, and 25% of the included studies adequately excluded bias in the selection of objective measures, while 75% excluded bias in self-reported outcomes. Overall, only two of the four studies were shown to have a low risk of reporting bias.

## 4. Discussion

Three out of the four studies analyzed in this systematic review are in agreement that there is a correlation between stress and anxiety related to the COVID-19 period and aggravation or onset of typical TMD symptoms. The mechanisms are neurological and complex, only some neuronal mechanisms involved in this have been hypothesized. Certainly, anxiety is increased due to the uncertainty of timing and lack of social relationships. Therefore, it can be said that the clinician should take this into consideration when making a treatment plan for patients with this COVID-19 anxiety- and stress-related TMD. These results support, the already hotly contested notion in the literature, that emotion plays a significant role in the development and progression of TMD. They also highlight the fact that pain management and control strategies can have a positive impact on symptoms of anxiety. The symptoms of temporomandibular dysfunction are complex and include both emotional changes as well as physical symptoms, with pain serving as the primary impetus for seeking therapy (for example, anxiety). A behavioral remedy should be used as a result. These patients can be managed with the help of the treatment methods employed here, which have shown promising short-term outcomes. The studies examined here evaluated the possible influence of COVID-19 on the onset and aggravation of temporomandibular symptoms. Three of the four studies reviewed assessed how there was an increase in anxiety and stress given by COVID-19, all of which were assessed through questionnaires. The COVID-19 era has enabled the development and implementation of new therapeutic techniques, which together with classic occlusal therapy, serve to improve symptoms and anxiety status, augmented by COVID-19. These techniques are the self-massage of tense and painful areas, stretching, thermotherapy, drug therapy, relaxation techniques, meditation, and mindfulness. All these factors can lead to the aggravation of TMD symptoms. Throughout human history, several pandemics have been addressed, such as the Black Death, tuberculosis, Spanish flu, and HIV/AIDS. However, the COVID-19 pandemic was the worst in contemporary history. Therefore, several studies have been carried out that related to the aggravation of TMD symptoms and the pandemic owing to a generalized increase in stress and anxiety due to the uncertainty about the future [55]. The underlying mechanism through which depressive symptoms and painful TMD are related by COVID-19 is more of a hypothesis than a foregone conclusion. Patients with physical pain frequently experience anxiety and depression, which typically accounts for neuroplastic alterations in the central nervous system. The central nervous system’s diminished ability to produce monoamine neurotransmitters such as 5-hydroxytryptamine and norepinephrine may contribute to depression. Monoamine neurotransmitters play a significant role in the initiation and progression of pain. The development and occurrence of chronic pain and depression have also been linked to glutamate and its receptor subtypes. Additionally, the surrounding inflammatory reaction contributes to both pain and sadness; thus, pain caused by the inflammatory response may be more strongly linked to depression. Neurotransmitter metabolism, neuroendocrine functioning, and neuroplasticity can all change as a result of inflammatory signals. Expression of the brain-derived neurotrophic factor is also diminished in patients with impaired functions in the hippocampus, prefrontal cortex, and other depression-related brain regions. The impact of the COVID-19 pandemic on these neurophysiological alterations in patients with painful TMD requires more investigation [56]. Discomfort in the jaw muscles and joint area, as well as clicking or crunching sounds when talking, chewing, yawning, or opening or closing the mouth, are some of the most typical symptoms of temporomandibular disorders (TMDs). Headaches, neck pain, and soreness in the teeth or temples may all be symptoms of TMD. Stress and psychosocial impairment are two of the main causes of TMD, which reflects the masticatory system’s malfunction. Uncertainty about the virus’s origin and nature, as well as governments’ abilities to stop its spread, lack of faith in healthcare systems and their capacities to handle new infections and risks, inaccurate information, and the solitary and resentment experienced by people in isolation due to a lack of socialization, all play significant associations in the emergence and conservation of TMD [57]. These behavioral components, which are frequently linked to sympathetic activity and extra adrenocortical steroid release, may cause peripheral vascular resistance and muscle vasoconstriction. In addition to feeling similar to frost and hot temperatures, abdomen pain, tachycardia, nausea, palpitations, diarrhea, and autonomic insufficiency can enhance the sympathetic impulse and the impression of hyperexcitement, as well as constipation, which causes and sustain sleep disturbances. According to reports, more people have reported grinding their teeth and having oral pain as a result of heightened stress brought on by health concerns, job loss, lockdown conditions, and being apart from loved ones. On the other hand, COVID-19-related stress, worry, and depression worsen symptoms of TMD, bruxism, and orofacial discomfort. Further, new studies found that patients with TMD are more likely to experience COVID-19 distress, which worsens their psychological condition and increases the severity of their chronic facial pain. These findings support the idea that stress amplifies central sensitization, anxiety, depression, chronic pain, and pain-related disability in TMD cases. Similar to most public health problems, pandemics are stressful. Aspects of psychological responses to epidemics and pandemics are discussed in the literature and lean on vulnerabilities, intolerance for ambiguity, perceived disease vulnerability, and anxiety. TMD may result from the stress, anxiety, and despair that people experienced as a result of the COVID-19 epidemic. Few articles have been found in the literature that correlates temporomandibular disorders with COVID-19. Furthermore, several studies have concluded that socioeconomic factors, poverty, and gender are key factors and predispose the aggravation of temporomandibular symptoms. This pandemic situation has changed social relationships, as well as the lifestyle of the entire population, these are all factors that contribute to the well-being of an individual, likewise their mental and psychological health and mortality. The COVID-19 pandemic made social relations absent or almost completely nil, causing social isolation to become mandatory with the consequent repercussions on mental health. The WHO acknowledged that the absence of social and interpersonal relationships has exacerbated symptoms such as depression and anxiety [58]. The study by Wang et al. showed that during the pandemic, there was a considerable psychological impact on people’s anxiety and depression. Therefore, now we must also consider the repercussions of what this state of anxiety has on the physiology of individuals. It has been hypothesized that this state of anxiety and uncertainty may have repercussions on TMD symptoms and onset [59]. The National Institute of Dental and Craniofacial Research found that 15% of the global population suffers from TMD. It must be emphasized that TMDs are often not accompanied by pain, however, if pain occurs, this pathology can become very disabling for life and for daily activities. Additionally, many studies have claimed that many TMD patients suffered from orofacial pain after the pandemic, while others had increased pain intensity during the pandemic. All this is important for dentists who are faced with a somatization of the psychological symptoms of patients. Increase chronic pain related to TMD may also occur due to social isolation and the lack of dental care for several months [60]. The studies analyzed in this review agree that the lockdown period and uncertainty about the future have caused an increase and exacerbation of facial pain and disorders, such as bruxism. The studies considered have evaluated the painful symptoms in groups of patients with TMD and have observed how anxiety and stress from COVID-19 have led to an increase in TMD pain and onset. Only the Mendonca et al. study found no differences between orofacial pain, temporomandibular joint disorders, and COVID-19 [61]. We can certainly conclude that patients already suffering from chronic TMD during the pandemic period were more prone to exacerbation of their symptoms, while patients who did not have temporomandibular joint disorders are more prone to their onset. Therefore, dentists must be able to quickly intercept these symptoms early and intervene with appropriate therapies, even with a psychological approach to treat this disabling pathology. In fact, various studies have shown how the lifestyle questionnaire is much worse in TMD patients with acute pain and is even debilitating in everyday life.

## 5. Conclusions

All the reviewed studies agree that there is an association between COVID-19 and an increased incidence of temporomandibular disorders. This systematic review showed that the state of uncertainty in which people lived during this pandemic, especially through stress, created muscle hyperactivity, and aggravation of bruxism, which are all causal factors and symptoms of temporomandibular dysfunction. Therefore, the clinician must assess this new issue of COVID-19 anxiety and stress, and for this reason must also, if TMDs are related to this situation, intervene with appropriate therapies, which should be carried out by multidisciplinary teams, consisting of dentists, physicians, and psychologists. If there is a need, specific drug therapy acting on anxiety should be used. In fact, occlusal therapy alone would not be sufficient, which is why it was also necessary to investigate this topic in further depth in order to help the clinic in solving this joint problem.

Therefore, the best way is to intercept early the people most prone to this state of anxiety and intervene pharmacologically in order to be able to reduce their anxiety and sense of agitation over the uncertainty about the future.

## Figures and Tables

**Table 1:** Search strategy.

***PubMed*** Search: (temporomandibular disorder) AND (COVID) (“temporomandibular joint disorders” [MeSH Terms] OR (“temporomandibular” [All Fields] AND “joint” [All Fields] AND “disorders” [All Fields]) OR “temporomandibular joint disorders” [All Fields] OR (“temporomandibular” [All Fields] AND “disorder” [All Fields]) OR “temporomandibular disorder” [All Fields]) AND (“SARS-CoV-2” [MeSH Terms] OR “SARS-CoV-2” [All Fields] OR “COVID” [All Fields] OR “covid 19” [MeSH Terms] OR “COVID 19” [All Fields])
***Web of Science*** (ALL = (temporomandibular disorders)) AND ALL = (COVID)
***Lilacs*** temporomandibular disorders [Palavras] and COVID [Palavras]
***Scopus*** TITLE-ABS-KEY (temporomandibular AND disorders AND COVID)

**Table 2:** Summary of the analyzed data.

Authors	Year	Nationality	Type of Study	Classification of TMD	Samples	Age	Type and Severity of Symptoms	Main Findings	Difference between Pre and COVID Era
Winocur-Arias et al. [51]	2022	Israel	Retrospective	Axis I DC/TMD	288 patients were divided into two groups. Group 1: 108 patients pre-COVID era. Group 2: 180 patients COVID era	35.6 yr	Evaluation of myalgia, myofascial pain, parafunction, bruxism	The results of this study showed a significant increase in parafunction in both men and women in the COVID era	Increase symptoms of TMD during COVID era
Visco et al. [52]	2020	Italy	Randomized clinical trial	Axis II RDC/TMD	A group of 182 patients	45 yr	Evaluation of TMJ with AXIS II for evaluated TMD and stress with PSS (perceived stress scale)	A questionnaire was administered. This evaluates chronic jaw pain, the impediment of social life that follows pain, and the evaluation of psychosocial stress. 51.4% of these subjects reported that symptoms worsened following the lockdown	Increase symptoms of TMJ during COVID era
Mendonca et al. [53]	2022	Brazil	Randomized clinical trial	RDC/TMD	41 women with temporomandibular disease.	30 yr	The study compared pain intensity and oral health-related quality of life (OHRQoL) in women with temporomandibular disorder	The data were collected in two stages. At time T1, prior to the pandemic, and at time T2 during the pandemic period.). No statistically significant differences were found in pain (*p* = 0.26) and overall OHIP-14 scores	No statistical significance
Asquini et al. [54]	2020	Italy	Prospective cohort study	Axis II RDC/TMD	45 adults before the lockdown (19 chronic, 26 acute/subacute TMD). A Covid stress scale was administered during the lockdown	29 yr	Evaluation of TMD pre and after the lockdown with CSS to evaluate the possible stress during lockdown in the onset of TMJ	Scores on the scale were significantly higher in patients with chronic TMD (*p* < 0.05). In people with chronic TMD. The change in chronic pain was statistically correlated with the change in CSS scores (r = 0.59; *p* = 0.017)	COVID worsened pain in patients with chronic TMJ

## Data Availability

Not applicable.

## References

[1] Curci C., Pisano F., Bonacci E., Camozzi D.M., Ceravolo C., Bergonzi R., De Franceschi S., Moro P., Guarnieri R., Ferrillo M. (2020). Early rehabilitation in post-acute COVID-19 patients: Data from an Italian COVID-19 rehabilitation unit and proposal of a treatment protocol. Eur. J. Phys. Rehabil. Med..

[2] Bennardo F., Buffone C., Fortunato L., Giudice A. (2020). COVID-19 is a challenge for dental education—A commentary. Eur. J. Dent. Educ..

[3] Badnjević A., Pokvić L.G., Džemić Z., Bečić F. (2020). Risks of emergency use authorizations for medical products during outbreak situations: A COVID-19 case study. Biomed. Eng. Online.

[4] Machoň V., Levorová J., Beňo M., Foltán R. (2022). The Manifestations of Covid-19 Infection. Manifestations in Patients with Temporomandibular Joint Disorders. Prague Med. Rep..

[5] Haddad C., Sayegh S.M., El Zoghbi A., Lawand G., Nasr L. (2022). The Prevalence and Predicting Factors of Temporomandibular Disorders in COVID-19 Infection: A Cross-Sectional Study. Cureus.

[6] Badnjević A., Cifrek M., Koruga D. Integrated software suite for diagnosis of respiratory diseases. Proceedings of the Eurocon 2013.

[7] Badnjevic A., Koruga D., Cifrek M., Smith H.J., Bego T. Interpretation of pulmonary function test results in relation to asthma classification using integrated software suite. 2013 36th International Convention on Information and Communication Technology, Electronics and Microelectronics (MIPRO).

[8] Granulo E., Bećar L., Gurbeta L., Badnjevic A. (2016). Telemetry System for Diagnosis of Asthma and Chronical Obstructive Pulmonary Disease (COPD). The Internet of Things Technologies for HealthCare, Västerås, Sweden, 18–19 October 2016.

[9] Stokes K., Castaldo R., Franzese M., Salvatore M., Fico G., Pokvic L.G., Badnjevic A., Pecchia L. (2021). A machine learning model for supporting symptom-based referral and diagnosis of bronchitis and pneumonia in limited resource settings. Biocybern. Biomed. Eng..

[10] Nahidh M., Al-Khawaja N.F.K., Jasim H.M., Cervino G., Cicciù M., Minervini G. (2023). The Role of Social Media in Communication and Learning at the Time of COVID-19 Lockdown—An Online Survey. Dent. J..

[11] de Medeiros R.A., Vieira D.L., da Silva E.V.F., de Rezende L.V.M.L., dos Santos R.W., Tabata L.F. (2020). Prevalence of symptoms of temporomandibular disorders, oral behaviors, anxiety, and depression in dentistry students during the period of social isolation due to COVID-19. J. Appl. Oral Sci..

[12] Emodi-Perlman A., Eli I. (2021). One year into the COVID-19 pandemic—Temporomandibular disorders and bruxism: What we have learned and what we can do to improve our manner of treatment. Dent. Med. Probl..

[13] Vrbanović E., Alajbeg I.Z., Alajbeg I. (2020). COVID-19 pandemic and Zagreb earthquakes as stressors in patients with temporomandibular disorders. Oral Dis..

[14] La Torre G., Shivkumar S., Mehta V., Kumar Vaddamanu S., Shetty U.A., Hussain Alhamoudi F., Ali Alwadi M.M., Ibrahim Aldosari L.N., Ali Alshadidi A.F., Minervini G. (2023). Surgical Protocols before and after COVID-19-A Narrative Review. Vaccines.

[15] Bennardo F., Antonelli A., Barone S., Figliuzzi M.M., Fortunato L., Giudice A. (2020). Change of Outpatient Oral Surgery during the COVID-19 Pandemic: Experience of an Italian Center. Int. J. Dent..

[16] Sycinska-Dziarnowska M., Maglitto M., Woźniak K., Spagnuolo G. (2021). Oral Health and Teledentistry Interest during the COVID-19 Pandemic. J. Clin. Med..

[17] Campus G., Diaz-Betancourt M., Cagetti M., Carvalho J., Carvalho T., Cortés-Martinicorena J., Deschner J., Douglas G., Giacaman R., Machiulskiene V. (2020). Study Protocol for an Online Questionnaire Survey on Symptoms/Signs, Protective Measures, Level of Awareness and Perception Regarding COVID-19 Outbreak among Dentists. A Global Survey. Int. J. Environ. Res. Public Health.

[18] Lee G.S., Kim H.K., Kim M.E. (2022). Relevance of sleep, pain cognition, and psychological distress with regard to pain in patients with burning mouth syndrome. Cranio J. Craniomandib. Pract..

[19] Liu M., Liu M., Lv K. (2020). Recommended Management of Temporomandibular Joint Dislocation During the COVID-19 Outbreak. J Craniofac. Surg..

[20] Cicciù M., Laino L., Fiorillo L. (2020). Oral signs and symptoms of COVID-19 affected patients: Dental practice as prevention method. Minerva Dent. Oral Sci..

[21] Troiano G., Dioguardi M., Giannatempo G., Laino L., Testa N.F., Cocchi R., De Lillo A., Muzio L.L. (2015). Orofacial Granulomatosis: Clinical Signs of Different Pathologies. Med. Princ. Pract..

[22] Fiorillo L., De Stefano R., Cervino G., Crimi S., Bianchi A., Campagna P., Herford A.S., Laino L., Cicciù M. (2019). Oral and Psychological Alterations in Haemophiliac Patients. Biomedicines.

[23] Cicciù M., Herford A.S., Cervino G., Troiano G., Lauritano F., Laino L. (2017). Tissue Fluorescence Imaging (VELscope) for Quick Non-Invasive Diagnosis in Oral Pathology. J. Craniofacial Surg..

[24] Straburzyński M., Nowaczewska M., Budrewicz S., Waliszewska-Prosół M. (2022). COVID-19-related headache and sinonasal inflammation: A longitudinal study analysing the role of acute rhinosinusitis and ICHD-3 classification difficulties in SARS-CoV-2 infection. Cephalalgia.

[25] Daltaban Ö., Aytekin Z.A. (2022). Fear and anxiety of COVID-19 in dental patients during the COVID-19 pandemic: A cross-sectional survey in Turkey. Dent. Med. Probl..

[26] Martina S., Amato A., Faccioni P., Iandolo A., Amato M., Rongo R. (2021). The perception of COVID-19 among Italian dental patients: An orthodontic point of view. Prog. Orthod..

[27] Franco R., Basili M., Venditti A., Chiaramonte C., Ottria L., Barlattani A., Bollero P. (2016). Statistical analysis of the frequency distribution of signs and symptoms of patients with temporomandibular disorders. Oral Implant..

[28] Emodi-Perlman A., Eli I., Smardz J., Uziel N., Wieckiewicz G., Gilon E., Grychowska N., Wieckiewicz M. (2020). Temporomandibular Disorders and Bruxism Outbreak as a Possible Factor of Orofacial Pain Worsening during the COVID-19 Pandemic—Concomitant Research in Two Countries. J. Clin. Med..

[29] Gaş S., Özsoy H.E., Aydın K.C. (2021). The association between sleep quality, depression, anxiety and stress levels, and temporomandibular joint disorders among Turkish dental students during the COVID-19 pandemic. Cranio^®^ J. Craniomandib. Pract..

[30] Rathi S., Chaturvedi S., Abdullah S., Rajput G., Alqahtani N.M., Chaturvedi M., Gurumurthy V., Saini R., Bavabeedu S.S., Minervini G. (2023). Clinical Trial to Assess Physiology and Activity of Masticatory Muscles of Complete Denture Wearer Following Vitamin D Intervention. Medicina.

[31] Crescente G., Minervini G., Spagnuolo C., Moccia S. (2022). *Cannabis* Bioactive Compound-Based Formulations: New Perspectives for the Management of Orofacial Pain. Molecules.

[32] Minervini G., Fiorillo L., Russo D., Lanza A., D’Amico C., Cervino G., Meto A., Di Francesco F. (2022). Prosthodontic Treatment in Patients with Temporomandibular Disorders and Orofacial Pain and/or Bruxism: A Review of the Literature. Prosthesis.

[33] Di Francesco F., Lanza A., Di Blasio M., Vaienti B., Cafferata E.A., Cervino G., Cicciù M., Minervini G. (2022). Application of Botulinum Toxin in Temporomandibular Disorders: A Systematic Review of Randomized Controlled Trials (RCTs). Appl. Sci..

[34] Minervini G.D., D’Amico C.D., Cicciù M.D., Fiorillo L.D. (2022). Temporomandibular Joint Disk Displacement: Etiology, Diagnosis, Imaging, and Therapeutic Approaches. J. Craniofacial Surg..

[35] Minervini G., Mariani P., Fiorillo L., Cervino G., Cicciù M., Laino L. (2022). Prevalence of temporomandibular disorders in people with multiple sclerosis: A systematic review and meta-analysis. Cranio^®^.

[36] Minervini G.D., Del Mondo D.D., Russo D.D., Cervino G.D., D’Amico C.D., Fiorillo L.D. (2022). Stem Cells in Temporomandibular Joint Engineering: State of Art and Future Persectives. J. Craniofacial Surg..

[37] Giudice A., Barone S., Muraca D., Averta F., Diodati F., Antonelli A., Fortunato L. (2020). Can Teledentistry Improve the Monitoring of Patients during the Covid-19 Dissemination? A Descriptive Pilot Study. Int. J. Environ. Res. Public Health.

[38] Chakraborty T., Jamal R., Battineni G., Teja K., Marto C., Spagnuolo G. (2021). A Review of Prolonged Post-COVID-19 Symptoms and Their Implications on Dental Management. Int. J. Environ. Res. Public Health.

[39] Spagnuolo G., De Vito D., Rengo S., Tatullo M. (2020). COVID-19 Outbreak: An Overview on Dentistry. Int. J. Environ. Res. Public Health.

[40] Reddy L.K.V., Madithati P., Narapureddy B.R., Ravula S.R., Vaddamanu S.K., Alhamoudi F.H., Minervini G., Chaturvedi S. (2022). Personalized Medicine Perception about Health Applications (Apps) in Smartphones towards Telemedicine during COVID-19: A Cross-Sectional Study. J. Pers. Med..

[41] Qazi N., Pawar M., Padhly P.P. (2023). 2023 Teledentistry: Evaluation of Instagram posts related to Bruxism. Technol. Health Care.

[42] Vlăduțu D., Popescu S.M., Mercuț R., Ionescu M., Scrieciu M., Glodeanu A.D., Stănuși A., Rîcă A.M., Mercuț V. (2022). Associations between Bruxism, Stress, and Manifestations of Temporomandibular Disorder in Young Students. Int. J. Environ. Res. Public Health.

[43] Cavallo L., Marcianò A., Cicciù M., Oteri G. (2020). 3D Printing beyond Dentistry during COVID 19 Epidemic: A Technical Note for Producing Connectors to Breathing Devices. Prosthesis.

[44] Scarano A., Inchingolo F., Lorusso F. (2020). Environmental Disinfection of a Dental Clinic during the Covid-19 Pandemic: A Narrative Insight. BioMed. Res. Int..

[45] Charitos I.A., Del Prete R., Inchingolo F., Mosca A., Carretta D., Ballini A., Santacroce L. (2020). What we have learned for the future about COVID-19 and healthcare management of it?. Acta Biomed..

[46] Colonna A., Guarda-Nardini L., Ferrari M., Manfredini D. (2021). COVID-19 pandemic and the psyche, bruxism, temporomandibular disorders triangle. Cranio^®^ J. Craniomandib. Pract..

[47] Straburzyński M., Kuca-Warnawin E., Waliszewska-Prosół M. (2022). COVID-19-related headache and innate immune response—A narrative review. Neurol. Neurochir. Pol..

[48] Peixoto K.O., de Resende C.M.B.M., de Almeida E.O., Almeida-Leite C.M., Conti P.C.R., Barbosa G.A.S., Barbosa J.S. (2021). Association of sleep quality and psychological aspects with reports of bruxism and TMD in Brazilian dentists during the COVID-19 pandemic. J. Appl. Oral Sci..

[49] Delgado-Rodríguez M., Sillero-Arenas M. (2018). Systematic review and meta-analysis. Med. Intensiva.

[50] Page M.J., McKenzie J.E., Bossuyt P.M., Boutron I., Hoffmann T.C., Mulrow  C.D., Shamseer L., Tetzlaff J.M., Akl E.A., Brennan S.E. (2021). The PRISMA 2020 statement: An updated guideline for reporting systematic reviews. Int. J. Surg..

[51] Winocur-Arias O., Winocur E., Shalev-Antsel T., Reiter S., Shifra L., Emodi-Perlman A., Friedman-Rubin P. (2022). Painful Temporomandibular Disorders, Bruxism and Oral Parafunctions before and during the COVID-19 Pandemic Era: A Sex Comparison among Dental Patients. J. Clin. Med..

[52] Saccomanno S., Bernabei M., Scoppa F., Pirino A., Mastrapasqua R., Visco M.A. (2020). Coronavirus Lockdown as a Major Life Stressor: Does It Affect TMD Symptoms?. Int. J. Environ. Res. Public Health.

[53] Mendonça A.K.R., Fontoura L.P.G., da Rocha T.D., Fontenele R.C., Nunes T.N.B., Regis R.R., Pinto-Fiamengui L.M.S. (2022). Influence of the COVID-19 pandemic on pain and oral health-related quality of life in women with temporomandibular disorder. Dent. Press J. Orthod..

[54] Asquini G., Bianchi A.E., Borromeo G., Locatelli M., Falla D. (2021). The impact of COVID-19-related distress on general health, oral behaviour, psychosocial features, disability and pain intensity in a cohort of Italian patients with temporomandibular disorders. PLoS ONE.

[55] Almeida-Leite C.M., Stuginski-Barbosa J., Conti P.C.R. (2020). How psychosocial and economic impacts of COVID-19 pandemic can interfere on bruxism and temporomandibular disorders?. J. Appl. Oral Sci..

[56] Perrone M.A., Feola A., Pieri M., Donatucci B., Salimei C., Lombardo M., Perrone A., Parisi A. (2021). The Effects of Reduced Physical Activity on the Lipid Profile in Patients with High Cardiovascular Risk during COVID-19 Lockdown. Int. J. Environ. Res. Public Health.

[57] Perrone M.A., Spolaore F., Ammirabile M., Romeo F., Caciagli P., Ceriotti F., Bernardini S. (2021). The assessment of high sensitivity cardiac troponin in patients with COVID-19: A multicenter study. IJC Heart Vasc..

[58] Scelza G., Amato A., Rongo R., Nucci L., D’Ambrosio F., Martina S. (2022). Changes in COVID-19 Perception and in TMD Prevalence after 1 Year of Pandemic in Italy. Eur. J. Dent..

[59] Gębska M., Dalewski B., Pałka Ł., Kołodziej Ł., Sobolewska E. (2021). The Importance of Type D Personality in the Development of Temporomandibular Disorders (TMDs) and Depression in Students during the COVID-19 Pandemic. Brain Sci..

[60] Lee Y.-H., Auh Q.-S. (2022). Clinical factors affecting depression in patients with painful temporomandibular disorders during the COVID-19 pandemic. Sci. Rep..

[61] Carrillo-Diaz M., Ortega-Martínez A.R., Romero-Maroto M., González-Olmo M.J. (2021). Lockdown impact on lifestyle and its association with oral parafunctional habits and bruxism in a Spanish adolescent population. Int. J. Paediatr. Dent..

